# Assessment of COVID-19 as the Underlying Cause of Death Among Children and Young People Aged 0 to 19 Years in the US

**DOI:** 10.1001/jamanetworkopen.2022.53590

**Published:** 2023-01-30

**Authors:** Seth Flaxman, Charles Whittaker, Elizaveta Semenova, Theo Rashid, Robbie M. Parks, Alexandra Blenkinsop, H. Juliette T. Unwin, Swapnil Mishra, Samir Bhatt, Deepti Gurdasani, Oliver Ratmann

**Affiliations:** 1 Department of Computer Science, University of Oxford, Oxford, United Kingdom; 2 MRC Centre for Global Infectious Disease Analysis, Jameel Institute for Disease and Emergency Analytics, Imperial College London, United Kingdom; 3 Department of Epidemiology and Biostatistics, School of Public Health, Imperial College London, United Kingdom; 4 Department of Environmental Health Sciences, Mailman School of Public Health, Columbia University, New York, New York; 5 Department of Mathematics, Imperial College London, United Kingdom; 6 Department of Public Health, University of Copenhagen, Copenhagen, Denmark; 7 Saw Swee Hock School of Public Health, National University of Singapore, Singapore; 8 Queen Mary University of London, United Kingdom

## Abstract

**Importance:**

COVID-19 was the underlying cause of death for more than 940 000 individuals in the US, including at least 1289 children and young people (CYP) aged 0 to 19 years, with at least 821 CYP deaths occurring in the 1-year period from August 1, 2021, to July 31, 2022. Because deaths among US CYP are rare, the mortality burden of COVID-19 in CYP is best understood in the context of all other causes of CYP death.

**Objective:**

To determine whether COVID-19 is a leading (top 10) cause of death in CYP in the US.

**Design, Setting, and Participants:**

This national population-level cross-sectional epidemiological analysis for the years 2019 to 2022 used data from the US Centers for Disease Control and Prevention Wide-Ranging Online Data for Epidemiologic Research (WONDER) database on underlying cause of death in the US to identify the ranking of COVID-19 relative to other causes of death among individuals aged 0 to 19 years. COVID-19 deaths were considered in 12-month periods between April 1, 2020, and August 31, 2022, compared with deaths from leading non–COVID-19 causes in 2019, 2020, and 2021.

**Main Outcomes and Measures:**

Cause of death rankings by total number of deaths, crude rates per 100 000 population, and percentage of all causes of death, using the National Center for Health Statistics 113 Selected Causes of Death, for ages 0 to 19 and by age groupings (<1 year, 1-4 years, 5-9 years, 10-14 years, 15-19 years).

**Results:**

There were 821 COVID-19 deaths among individuals aged 0 to 19 years during the study period, resulting in a crude death rate of 1.0 per 100 000 population overall; 4.3 per 100 000 for those younger than 1 year; 0.6 per 100 000 for those aged 1 to 4 years; 0.4 per 100 000 for those aged 5 to 9 years; 0.5 per 100 000 for those aged 10 to 14 years; and 1.8 per 100 000 for those aged 15 to 19 years. COVID-19 mortality in the time period of August 1, 2021, to July 31, 2022, was among the 10 leading causes of death in CYP aged 0 to 19 years in the US, ranking eighth among all causes of deaths, fifth in disease-related causes of deaths (excluding unintentional injuries, assault, and suicide), and first in deaths caused by infectious or respiratory diseases when compared with 2019. COVID-19 deaths constituted 2% of all causes of death in this age group.

**Conclusions and Relevance:**

The findings of this study suggest that COVID-19 was a leading cause of death in CYP. It caused substantially more deaths in CYP annually than any vaccine-preventable disease historically in the recent period before vaccines became available. Various factors, including underreporting and not accounting for COVID-19’s role as a contributing cause of death from other diseases, mean that these estimates may understate the true mortality burden of COVID-19. The findings of this study underscore the public health relevance of COVID-19 to CYP. In the likely future context of sustained SARS-CoV-2 circulation, appropriate pharmaceutical and nonpharmaceutical interventions (eg, vaccines, ventilation, air cleaning) will continue to play an important role in limiting transmission of the virus and mitigating severe disease in CYP.

## Key Points

**Question:**

Where does COVID-19 rank as an underlying cause of death for children and young people aged 0 to 19 years in the US?

**Findings:**

Among children and young people aged 0 to 19 years in the US, COVID-19 ranked eighth among all causes of deaths, fifth in disease-related causes of deaths (excluding unintentional injuries, assault, and suicide), and first in deaths caused by infectious or respiratory diseases. COVID-19 deaths constituted 2% of all causes of death in this age group.

**Meaning:**

In this study, COVID-19 posed a significant disease burden for children and young people, so pharmaceutical and nonpharmaceutical interventions continue to be important to limit transmission of the virus and to mitigate severe disease.

## Teaser

This cross-sectional study evaluates whether COVID-19 is among the top 10 causes of death in children and young people aged 0 to 19 years in the US.

## Introduction

In the 12-month period August 1, 2021, to July 31, 2022, there were more than 360 000 deaths from COVID-19 in the US^1^ (a rate of 109 per 100 000 population). In children and young people (CYP) aged 0 to 19 years, there were 821 deaths from COVID-19 reported in this time period (1.0 per 100 000 population). The overall risk of death from COVID-19 in CYP is thus substantially less than in other age groups in the US. However, deaths in US CYP from all causes are rare (49.4 per 100 000 in 2019 for those aged 0-19 years; 25.0 per 100 000 for those aged 1 to 19 years), and so the mortality burden of COVID-19 is best understood by comparing it with other significant causes of CYP mortality from a recent pre–COVID-19 period. For this purpose, we used the US Centers for Disease Control and Prevention (CDC) Wide-Ranging Online Data for Epidemiologic Research (WONDER) database of mortality statistics on Underlying Cause of Death. Rankable causes of death are defined by the US National Center for Health Statistics (NCHS)’s grouping of the 113 Selected Causes of Death mortality tabulation list of *International Statistical Classification of Diseases and Related Health Problems, Tenth Revision *(*ICD-10*) codes for underlying cause of death. The Selected Causes of Death were originally drawn up in 1951 to allow comparisons for public health purposes and are regularly updated,^2^ with COVID-19 (*ICD-10-CM* code U07.1) added to this list in October 2020.^3^ Leading causes of death are one of various ways of understanding mortality and burden of disease. They are a starting point for a high-level understanding of public health priorities and resource allocation. We considered the 10 leading causes of death in 2019, that is, the ordered list (1st through 10th) of causes that occur most frequently among NCHS’s rankable causes of death.^2^ We assessed COVID-19 as the underlying cause of death among CYP aged 0 to 19 years in the US, determined whether it was among the 10 leading causes of death for CYP, and reported crude death rates per 100 000 population and the percentage of all deaths by rankable underlying cause of death.

## Methods

We followed the Strengthening the Reporting of Observational Studies in Epidemiology (STROBE) reporting guidelines for cross-sectional studies. For ethics, we used the Health Research Authority decision tools.^4^ Our study was considered research, and according to the NHS Research Ethics Committee review tool,^5^ we did not need NHS Research Ethics Committee review or informed consent, as we only used (1) publicly available, (2) anonymized, and (3) aggregated data outside of clinical settings.

### Statistical Analysis

We obtained the 10 leading causes of death among the rankable groupings of underlying causes of death from the NCHS 113 Selected Causes of Death by age group in 2019 from CDC WONDER,^6^ comprehensively described elsewhere.^2^ We compared these with COVID-19 mortality as an underlying cause of death in our study period, August 1, 2021, to July 31, 2022, obtained from CDC WONDER Provisional Mortality Statistics^1^ (the most recent 12-month period in which data are close to complete^7^). We chose the most recent period as this is likely to be representative of continuing widespread circulation of Omicron subvariants, availability of vaccines, and limited nonpharmaceutical interventions. However, we acknowledge that no study period is without limitations, so we also carried out sensitivity analyses for all possible 12-month periods between April 2020 to August 2022 to examine consistency in ranking over time. As further sensitivity analyses, we considered data from CDC WONDER on leading causes of death in 2015 to 2019 (in case 2019 was an outlier) and data from CDC WONDER on leading causes of death in 2020 and 2021. An underlying cause of death is defined as a disease or injury that initiates a series of events leading directly to death. To determine pediatric death rates by cause of death, we used 2019 and 2021 population size estimates by single year of age from the US Census Bureau^8^ and calculated crude death rates by dividing reported deaths in a given age group with the corresponding population size estimates. We report crude death rates per 100 000 population rather than per case, meaning that we are estimating the total burden in the population rather than case fatality or infection fatality ratios.

We note that NCHS’s list of rankable causes of death usually group together many individual *ICD* codes,^2^ but COVID-19 is considered as a cause of death for a single *ICD-10-CM* code, U07.1—COVID-19 (U07.2 was not adopted in the US^9^). Thus, we are comparing the underlying cause of death from a single pathogen (SARS-CoV-2) to groupings of multiple underlying causes of death (such as influenza and pneumonia). By definition, underlying cause of death statistics do not include deaths in which COVID-19 was considered to have been a contributing cause of death,^1^ and thus differ from other data sets reporting COVID-19 deaths, eg, NCHS has a separate source reporting counts of deaths involving COVID-19^10^ (a previous, preprinted version of this study used these data^11^). We also note that the CDC WONDER Provisional Mortality Statistics could still be revised in the future. Statistical analyses were carried out with R version 4.1.2 (R Project for Statistical Computing).

## Results

**Figure 1.**
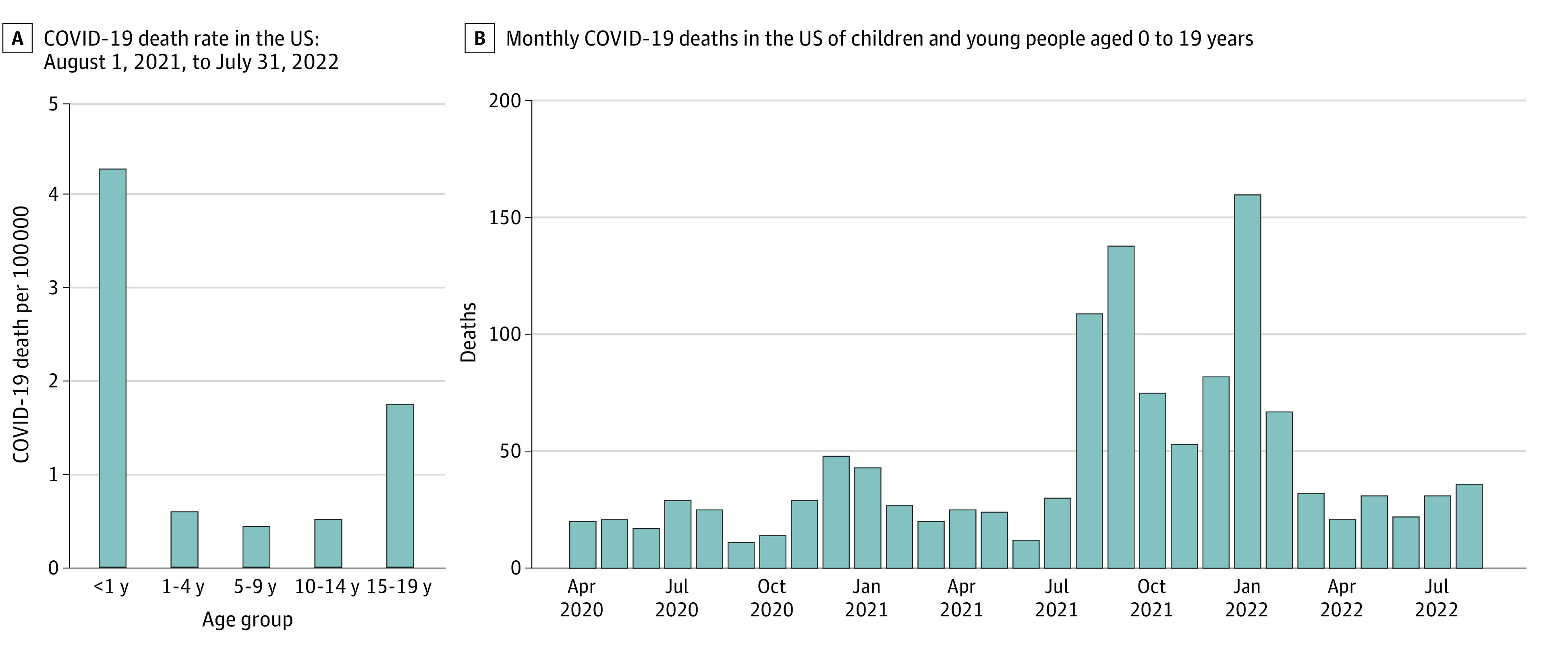
COVID-19 Deaths Among Children and Young People Aged 0 to 19 Years in the US A, COVID-19 death rates in the US for children and young people, where COVID-19 is listed as the underlying cause of death (*International Statistical Classification of Diseases and Related Health Problems, Tenth Revision *code U07.1) on the death certificate.^1^ Rates are calculated as COVID-19 deaths for the period August 1, 2021, to July 31, 2022, per 100 000 population (2021 population estimates). B, Monthly COVID-19 deaths in the US of children and young people, where COVID-19 is listed as the underlying cause of death (*International Statistical Classification of Diseases and Related Health Problems, Tenth Revision *code U07.1) on the death certificate.^1^

There were 82 million CYP aged 0 to 19 years in the US in 2021. In the study period, August 1, 2021, to July 31, 2022, there were 821 deaths in this age group for which COVID-19 was the underlying cause of death, for a crude death rate of 1.0 per 100 000 population. Pediatric COVID-19 death rates followed a U-shaped curve across age groups in the US (Figure 1A), a commonly observed pattern.^12,13^ We considered 5 age brackets: younger than 1 year, 1 to 4 years, 5 to 9 years, 10 to 14 years, and 15 to 19 years. In the study period, COVID-19 death rates in infants younger than 1 year were 4.3 deaths per 100 000 population, 0.6 per 100 000 in children aged 1 to 4 years, 0.4 per 100 000 in children aged 5 to 9 years, and 0.5 per 100 000 in children aged 10 to 14 years, increasing to 1.8 per 100 000 in those aged 15 to 19 years.

In 2019, leading causes of CYP deaths (Table 1) were perinatal conditions (12.7 per 100 000), unintentional injuries (9.1 per 100 000), congenital malformations or deformations (6.5 per 100 000), assault (3.4 per 100 000), suicide (3.4 per 100 000), malignant neoplasms (2.1 per 100 000), diseases of the heart (1.1 per 100 000), and influenza and pneumonia (0.6 per 100 000). For comparison, in the study period, August 1, 2021, to July 31, 2022, there were 821 CYP deaths reported for which the underlying cause was COVID-19 (1.0 per 100 000), meaning COVID-19 ranked as the eighth leading cause of death (Table 1) and accounted for 2.0% of all causes of death. Rankings disaggregated by age group are shown in eTable 1 in Supplement 1. COVID-19 ranked consistently in the top 10 leading causes of death in CYP in all age groups: seventh among those younger than 1 year; seventh among those aged 1 to 4 years; sixth among those aged 5 to 9 years; sixth among those aged 10 to 14 years; and fifth among those aged 15 to 19 years. COVID-19 accounted for 0.7% of deaths among those younger than 1 year; 2.5% among those aged 1 to 4 years; 3.8% among those aged 5 to 9 years; 3.5% among those aged 10 to 14 years; and 3.7% among those aged 15 to 19 years of all causes of death by age group.

**Table 1.** Deaths Among Individuals Aged 0 to 19 Years

Leading causes of death (*ICD-10* codes)^a^	Crude rate per 100 000	Deaths, No.	Rank	% Of all causes
#Certain conditions originating in the perinatal period (P00-P96)	12.7	10 387	1	25.7
#Accidents (unintentional injuries) (V01-X59, Y85-Y86)	9.1	7444	2	18.4
#Congenital malformations, deformations, and chromosomal abnormalities (Q00-Q99)	6.5	5286	3	13.1
#Assault (homicide) (*U01-*U02, X85-Y09, Y87.1)	3.4	2770	4	6.9
#Intentional self-harm (suicide) (*U03, X60-X84, Y87.0)	3.4	2756	5	6.8
#Malignant neoplasms (C00-C97)	2.1	1704	6	4.2
#Diseases of heart (I00-I09, I11, I13, I20-I51)	1.1	867	7	2.1
#COVID-19 (U07.1)	1.0	821	8	2.0
#Influenza and pneumonia (J09-J18)	0.6	472	9	1.2
#Cerebrovascular diseases (I60-I69)	0.4	297	10	0.7

^a^ Leading causes of death from the rankable causes on the National Center for Health Statistics 113 Selected Causes of Death List, for children and young people aged 0 to 19 years in 2019 in the US ranked, compared with COVID-19 deaths (August 1, 2021-July 31, 2022). COVID-19 was the eighth leading cause of death, and the fifth leading cause of death in disease-related causes of deaths (excluding unintentional injuries, assault, and suicide). The National Center for Health Statistics 113 Selected Causes of Death can be grouped into rankable causes of death, indicated by the # symbol. The * symbol indicates that U01-U03 are not *ICD-10* codes but were introduced by NCHS in 2001 to classify deaths due to acts of terrorism.

Excluding causes of death unrelated to disease (unintentional injuries, assault, and suicide), COVID-19 ranked as the fifth leading cause of death in US CYP (Table 1). Considering infectious and respiratory diseases only, COVID-19 ranked as the top (first) cause of death in US CYP (Table 2), followed by influenza and pneumonia as the second leading cause; although we note that the causative agents of pneumonia may include multiple pathogens.

**Table 2.** Causes of Death Among Individuals Aged 0 to 19 Years: Certain Infectious and Parasitic Diseases and Diseases of the Respiratory System

Leading cause of death, certain infectious and parasitic diseases and diseases of the respiratory system (*ICD-10* code)^a^	No.		
	Crude rate (per 100 000)	Deaths, No.	Rank
#COVID-19 (U07.1)	1.0	821	1
#Influenza and pneumonia (J09-J18)	0.6	472	2
Other and unspecified infectious and parasitic diseases and their sequelae (A00, A05, A20-A36, A42-A44, A48-A49, A54-A79, A81-A82, A85.0-A85.1, A85.8, A86-B04, B06-B09, B25-B49, B55-B99, U07.1)	0.5	432	3
Other diseases of respiratory system (J00-J06, J30- J39, J67, J70-J98)	0.5	421	4
Pneumonia (J12-J18)	0.4	300	5
#Septicemia (A40-A41)	0.4	287	6
#Chronic lower respiratory diseases (J40-J47)	0.3	259	7
Certain other intestinal infections (A04, A07-A09)	0.3	223	8
Asthma (J45-J46)	0.3	206	9
Influenza (J09-J11)	0.2	172	10

^a^ Causes of death from the A00 to B99 (Certain infectious and parasitic diseases) and J00 to J98 (Diseases of the respiratory system) causes on the National Center for Health Statistics 113 Selected Causes of Death. Data are for children and young people aged 0 to 19 years in 2019 in the US ranked by number of deaths, compared with COVID-19 deaths (August 1, 2021-July 31, 2022). The National Center for Health Statistics 113 Selected Causes of Death can be grouped into rankable causes of death, indicated by the # symbol. Thus, categories overlap in the table, eg, pneumonia (*ICD-10* codes J12-J18) is a subset of the rankable cause #Influenza and pneumonia (J09-J18). COVID-19 was added as a rankable cause to the NCHS 113 Selected Causes of Death list in 2020.^3^

For consistency, we have used the NCHS 113 Selected Causes of Death (which was designed for ages 1 year and older) for all CYP age group disaggregations and combinations, rather than the NCHS 130 Selected Causes of Infant Death. Perinatal causes of death predominate for neonates, and thus, COVID-19 is not a top 10 cause of death in the first 28 days of life using the 130 Causes List (eTable 2A in Supplement 1). Restricting to the post-neonatal age group of 28 to 364 days using the 130 Causes List, COVID-19 was a top 10 leading cause of death (eTable 2B in Supplement 1).

**Figure 2.**
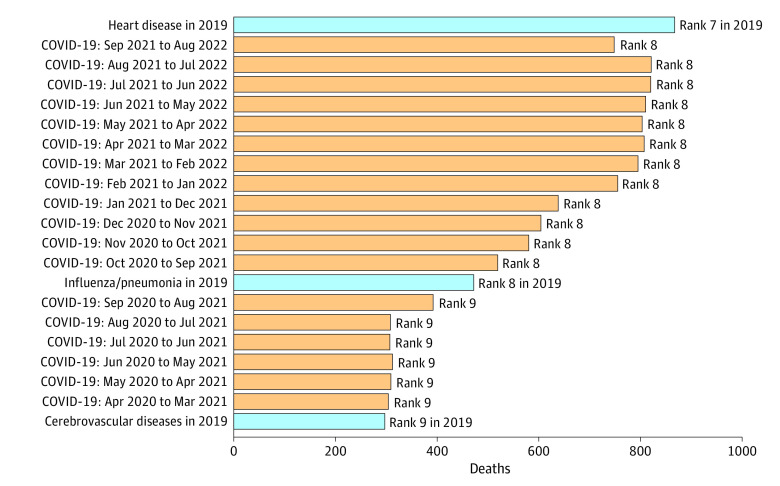
Leading Causes of Death in Children and Young People Compared With COVID-19 Deaths in Different 12 Month Periods For children and young people aged 0 to 19 years in 2019, leading causes of death included heart disease (ranked seventh), influenza/pneumonia (eighth), and cerebrovascular diseases (ninth). We compare these causes of death to COVID-19 deaths in each 12 month period for which data were available: April 2020 to March 2021, May 2020 to April 2021, and so on. Data for recent months are not yet complete.^14^

Our study period, August 1, 2021, to July 31, 2022, coincides with substantial infection waves of the COVID-19 Delta and Omicron variants (Figure 1B). As a sensitivity analysis, we considered every 12-month period from April 2020 to August 2022 (Figure 2).^14^ In the pre-Delta period, before July 2021, COVID-19 death rates were considerably lower than in the Delta and Omicron periods. Nevertheless, in the pre-Delta period, COVID-19 would have ranked as the ninth leading cause of death, rather than the eighth leading cause of death.

The 10 leading causes of death among CYP were largely unchanged when comparing 2019 with 2015 to 2019, with 2020, and with 2021, with the exception that in 2021 COVID-19 entered the top 10 as the eighth leading cause of death among CYP. This is the same rank we found for our study period (Table 1). As a final sensitivity analysis, had the 821 COVID-19 deaths in our study period occurred in 2015 to 2019 (annualized), 2020, or 2021 (3 separate comparisons as sensitivity analyses), COVID-19 would have ranked as the seventh or eighth leading cause of death in all 3 comparisons.

## Discussion

In this cross-sectional study of underlying causes of death among children aged 0 to 19 years in the US, we compared leading causes of death in 2019 with COVID-19 deaths. Early in the COVID-19 pandemic, comparisons of COVID-19 disease severity between age groups were a vital tool for appropriately allocating limited resources and prioritizing vaccination campaigns. However, long-term public health planning and management needs to be informed by the leading causes of deaths within each age group, a practice in the US dating back at least 7 decades, beginning with the first publication of a leading cause of death ranking in 1952.^2^ The most recent comprehensive prepandemic data on leading causes of death covers 2019.^2^ Compared with this period, we found that COVID-19 was a leading cause of death in CYP aged 0 to 19 years in the US for the period of August 1, 2021, to July 31, 2022, ranking eighth among all causes of deaths (representing 2.0% of all causes of death). Considering other 12-month periods during the pandemic did not qualitatively change our findings (Figure 2), nor did comparing with non–COVID-19 causes of death in other periods (ie, 2015-2019, 2020, and 2021).

While other causes of death, such as unintentional injuries (18.4%), assault (6.9%), and suicide (6.8%) represented a large percentage of all causes of death, COVID-19 ranked fifth in disease-related causes of deaths (excluding unintentional injuries, assault, and suicide), and first in deaths caused by infectious and respiratory diseases. Comparing deaths from COVID-19 with deaths from other vaccine-preventable diseases historically, COVID-19 caused substantially more deaths (821 deaths in our study period in CYP) than major vaccine-preventable diseases did before vaccines became available: hepatitis A (3 reported deaths in children per year in the US), rotavirus (20-60 reported deaths in children per year in the US), rubella (17 reported deaths in children per year in the US), varicella (50 reported deaths in children per year in the US),^15^ and measles (495 total reported deaths per year,^16^ the vast majority in children^17^).

In summary, we found that COVID-19 is now a leading cause of death for CYP aged 0-19 years in the US, and the top (first) leading cause of death among infectious and respiratory diseases. Overall, deaths in CYP increased over the Delta and Omicron waves compared with previous waves, likely reflecting the large numbers of CYP infected during these periods. Future variants (or subvariants) capable of displacing current Omicron subvariants will, by definition, have a growth advantage over these lineages, and there is no guarantee that their intrinsic severity will be reduced.^18^ In the context of sustained transmission and circulation of SARS-CoV-2 in the US, the nontrivial risk posed by COVID-19 to CYP warrants use of a wide and robust array of tools to limit the extent of infection and severe disease in this age group, through a combination of safe and efficacious vaccination against the disease,^19^ appropriate pharmaceutical treatments, mitigations such as ventilation,^20,21^ and other nonpharmaceutical interventions (eg, testing, supported isolation).

### Limitations

Our findings need to be considered in the context of several limitations which mean that we may have underestimated the true mortality burden of COVID-19 in CYP aged 0 to 19 years. Analyses of excess deaths have suggested underreporting bias in COVID-19 deaths^22^; specific criteria for determining COVID-19 deaths is heterogeneous across the US and has changed over time; and delays to reporting may be substantial for recent time periods.^14^ We consider COVID-19 as an underlying (and not contributing) cause of death only, but COVID-19 amplifies the severe impacts of other diseases, and mortality hazards from coinfection (eg, influenza^23^) are increased with accompanying comorbidities. The category of deaths from influenza and pneumonia combines a variety of causes, to which SARS-CoV-2 could be a contributing factor.^23,24^ Recent evidence also suggests that COVID-19 may contribute to serious long-term sequelae^25^ in children and adolescents, which are unlikely to have been captured in these data.

It is important to note that population death rates result from the combination of SARS-CoV-2 transmission rates and COVID-19 disease severity (infection fatality ratios), and both have varied significantly over the course of the pandemic. Transmission rates have varied due to changing nonpharmaceutical interventions (eg, school and business closures), behavior (eg, mask usage), and increasing population immunity from previous infection and vaccination. Varying population immunity also leads to lower infection fatality ratios. COVID-19 disease severity varies between variants^26,27^ with inconsistent findings for children vs older age groups.^27,28^ Nevertheless, our sensitivity analyses across time periods consistently showed COVID-19 to be a leading cause of death in CYP. We caution against using differences in COVID-19 death rates between periods to understand variant-specific severity given changes in viral transmission and disease severity.

## Conclusions

In this study, COVID-19 was a leading cause of death among individuals aged 0 to 19 years in the US. Our findings underscore the public health relevance of COVID-19 to CYP. In the likely future context of sustained SARS-CoV-2 circulation, appropriate pharmaceutical and nonpharmaceutical interventions (eg, vaccines, ventilation, air cleaning) will continue to play an important role in limiting transmission of the virus and mitigating severe disease in CYP.

## Notes

Supplement 1. **eTable 1. **Leading Causes of Death by Age Group **eTable 2.** Leading Causes of Death Among Those Younger than 28 Days, Aged 28 to 365 Days, and Aged 0 to 365 Days

Supplement 2.
Data Sharing Statement
