# Supplementary material for: Assessment of COVID-19 as the Underlying Cause of Death Among Children and Young People Aged 0 to 19 Years in the US
Source: JAMA Netw Open. 2023 Jan 30;6(1):e2253590. doi: 10.1001/jamanetworkopen.2022.53590 (PMC9887489; doi:10.1001/jamanetworkopen.2022.53590)
Supplement: Supplement 1. — eTable 1. Leading Causes of Death by Age Group eTable 2. Leading Causes of Death Among Those Younger than 28 Days, Aged 28 to 365 Days, and Aged 0 to 365 Days [file jamanetwopen-e2253590-s001.pdf]

Supplemental Online Content

Flaxman S, Whittaker C, Semenova E, et al. Assessment of COVID-19 as the underlying cause of death among children and young people aged 0 to 19 years in the US. *JAMA Netw Open.* 2023;6(1):e2253590. doi:10.1001/jamanetworkopen.2022.53590

eTable 1. Leading Causes of Death by Age Group

eTable 2. Leading Causes of Death Among Those Younger than 28 Days, Aged 28 to 365 Days, and Aged 0 to 365 Days

This supplemental material has been provided by the authors to give readers additional information about their work.

© 2023 Flaxman S et al. *JAMA Network Open.*

eTable 1. Leading Causes of Death by Age Group

eTable 1A<br>Age: <1 year				
**Leading Causes of Death**	**Crude Rate (per 100,000)**	**Deaths**	**Rank**	**% of all causes of death**
#Certain conditions originating in the perinatal period (P00-P96)	272.1	10294	1	49.2
#Congenital malformations, deformations and chromosomal abnormalities (Q00-Q99)	113.7	4301	2	20.6
#Accidents (unintentional injuries) (V01-X59,Y85-Y86)	33.5	1266	3	6.1
#Diseases of heart (I00-I09,I11,I13,I20-I51)	7.1	268	4	1.3
#Assault (homicide) (*U01-*U02,X85-Y09,Y87.1)	7	263	5	1.3
#Influenza and pneumonia (J09-J18)	4.1	156	6	0.7
#COVID-19 (U07.1)	4.3	153	7	0.7
#Septicemia (A40-A41)	3.5	133	8	0.6
#Cerebrovascular diseases (I60-I69)	2.7	102	9	0.5
#Nephritis, nephrotic syndrome and nephrosis (N00-N07,N17-N19,N25-N27)	1.6	61	10	0.3

© 2023 Flaxman S et al. *JAMA Network Open.*

eTable 1B<br>Age: 1-4 year olds				
**Leading Causes of Death**	**Crude Rate (per 100,000)**	**Deaths**	**Rank**	**% of all causes of death**
#Accidents (unintentional injuries) (V01-X59,Y85-Y86)	7.3	1149	1	31.3
#Congenital malformations, deformations and chromosomal abnormalities (Q00-Q99)	2.6	416	2	11.3
#Malignant neoplasms (C00-C97)	1.8	285	3	7.8
#Assault (homicide) (*U01-*U02,X85-Y09,Y87.1)	1.8	284	4	7.7
#Diseases of heart (I00-I09,I11,I13,I20-I51)	0.8	133	5	3.6
#Influenza and pneumonia (J09-J18)	0.8	122	6	3.3
#COVID-19 (U07.1)	0.6	91	7	2.5
#Certain conditions originating in the perinatal period (P00-P96)	0.4	57	8	1.6
#Septicemia (A40-A41)	0.3	53	9	1.4
#Cerebrovascular diseases (I60-I69)	0.3	52	10	1.4

© 2023 Flaxman S et al. *JAMA Network Open.*

eTable 1C<br>Age: 5-9 year olds				
**Leading Causes of Death**	**Crude Rate (per 100,000)**	**Deaths**	**Rank**	**% of all causes of death**
#Accidents (unintentional injuries) (V01-X59,Y85-Y86)	3.5	714	1	30.6
#Malignant neoplasms (C00-C97)	1.8	371	2	15.9
#Congenital malformations, deformations and chromosomal abnormalities (Q00-Q99)	1	192	3	8.2
#Assault (homicide) (*U01-*U02,X85-Y09,Y87.1)	0.8	155	4	6.6
#Diseases of heart (I00-I09,I11,I13,I20-I51)	0.5	91	5	3.9
#COVID-19 (U07.1)	0.4	89	6	3.8
#Chronic lower respiratory diseases (J40-J47)	0.3	69	7	3.0
#Influenza and pneumonia (J09-J18)	0.3	52	8	2.2
#Cerebrovascular diseases (I60-I69)	0.2	37	9	1.6
#Septicemia (A40-A41)	0.2	36	10	1.5

© 2023 Flaxman S et al. *JAMA Network Open.*

eTable 1D<br>Age: 10-14 year olds				
**Leading Causes of Death**	**Crude Rate (per 100,000)**	**Deaths**	**Rank**	**% of all cause s of death**
#Accidents (unintentional injuries) (V01-X59,Y85-Y86)	3.7	778	1	24.6
#Intentional self-harm (suicide) (*U03,X60-X84,Y87.0)	2.6	534	2	16.9
#Malignant neoplasms (C00-C97)	1.9	404	3	12.8
#Assault (homicide) (*U01-*U02,X85-Y09,Y87.1)	0.9	191	4	6.0
#Congenital malformations, deformations and chromosomal abnormalities (Q00-Q99)	0.9	189	5	6.0
#COVID-19 (U07.1)	0.5	110	6	3.5
#Diseases of heart (I00-I09,I11,I13,I20-I51)	0.4	87	7	2.7
#Chronic lower respiratory diseases (J40-J47)	0.4	81	8	2.6
#Influenza and pneumonia (J09-J18)	0.3	71	9	2.2
#Cerebrovascular diseases (I60-I69)	0.2	48	10	1.5

© 2023 Flaxman S et al. *JAMA Network Open.*

eTable 1E<br>Age: 15-19 year olds				
**Leading Causes of Death**	**Crude Rate (per 100,000)**	**Deaths**	**Rank**	**% of all causes of death**
#Accidents (unintentional injuries) (V01-X59,Y85-Y86)	16.8	3537	1	34.5
#Intentional self-harm (suicide) (*U03,X60-X84,Y87.0)	10.5	2210	2	21.5
#Assault (homicide) (*U01-*U02,X85-Y09,Y87.1)	8.9	1877	3	18.3
#Malignant neoplasms (C00-C97)	2.8	589	4	5.7
#COVID-19 (U07.1)	1.8	378	5	3.7
#Diseases of heart (I00-I09,I11,I13,I20-I51)	1.4	288	6	2.8
#Congenital malformations, deformations and chromosomal abnormalities (Q00-Q99)	0.9	188	7	1.8
#Influenza and pneumonia (J09-J18)	0.3	71	8	0.7
#Chronic lower respiratory diseases (J40-J47)	0.3	60	9	0.6
#Diabetes mellitus (E10-E14)	0.3	59	10	0.6

eTable 1. Leading causes of death from the rankable causes on the NCHS 113 Selected Causes of Death List, for children and young people aged 0-19 years in 2019 in the US ranked, compared to Covid-19 deaths (August 1, 2021-July 31, 2022). Deaths, crude rates per 100,000, ranks, and percentage of the 10 leading causes are shown for ages < 1 year (a), 1-4 years (b), 5-9 years (c), 10-14 years (d), 15-19 years (e). We used 2019 and 2021 population size estimates by single year of age from the US Census Bureau. In all cases, Covid-19 is among the 10 leading causes of death. (Versions of eTable 1A using the NCHS 130 Selected Causes of Infant Death are presented in eTables 2A-C. The predominance of perinatal causes of death means that in the first 28 days of life, Covid-19 is not a leading cause of death, but it is a top 10 cause of death from ages 28-364 days.)

© 2023 Flaxman S et al. *JAMA Network Open.*

eTable 2. Leading Causes of Death Among Those Younger than 28 Days, Aged 28 to 365 Days, and Aged 0 to 365 Days

**eTable 2A<br>Ages: < 28 days**			
**10 Leading Causes of Death (Infants)**	**Deaths**	**Crude Rate (per 1000 live births)**	**Rank**
#Disorders related to short gestation and low birth weight, not elsewhere classified (P07)	3,374	0.9	1
#Congenital malformations, deformations and chromosomal abnormalities (Q00-Q99)	3,047	0.8	2
#Newborn affected by maternal complications of pregnancy (P01)	1,240	0.3	3
#Newborn affected by complications of placenta, cord and membranes (P02)	731	0.2	4
#Bacterial sepsis of newborn (P36)	579	0.2	5
#Respiratory distress of newborn (P22)	409	0.1	6
#Neonatal hemorrhage (P50-P52,P54)	335	0.1	7
#Intrauterine hypoxia and birth asphyxia (P20-P21)	320	0.1	8
#Necrotizing enterocolitis of newborn (P77)	312	0.1	9
#Atelectasis (P28.0-P28.1)	230	0.1	10

Supplementary Table 2(a). Leading causes of death from the rankable causes on the NCHS 130 Selected Causes of Infant Death List, for neonates (< 28 days old) in 2019 in the US ranked. There were 35 Covid-19 deaths (August 1, 2021-July 31, 2022) for this age group. Deaths, crude rates per 1,000 live births, and ranks are shown. Source: https://wonder.cdc.gov/controller/saved/D76/D313F350

© 2023 Flaxman S et al. *JAMA Network Open.*

eTable 2B<br>Ages: 28-364 days			
**10 Leading Causes of Death (Infants)**	**Deaths**	**Crude Rate (per 1000 live births)**	**Rank**
#Congenital malformations, deformations and chromosomal abnormalities (Q00-Q99)	1,254	0.3	1
#Accidents (unintentional injuries) (V01-X59)	1,125	0.3	2
#Sudden infant death syndrome (R95)	1,110	0.3	3
#Diseases of the circulatory system (I00-I99)	326	0.1	4
#Assault (homicide) (*U01,X85-Y09)	247	0.1	5
#Diarrhea and gastroenteritis of infectious origin (A09)	180	0	6
#Influenza and pneumonia (J09-J18)	156	0	7
#Chronic respiratory disease originating in the perinatal period (P27)	148	0	8
#Septicemia (A40-A41)	131	0	9
#Disorders related to short gestation and low birth weight, not elsewhere classified (P07)	71	0	10

Supplementary Table 2(b). Leading causes of death from the rankable causes on the NCHS 130 Selected Causes of Infant Death List, for infants 28-364 days old in 2019 in the US ranked. There were 118 Covid-19 deaths (August 1, 2021-July 31, 2022) for this age group. Deaths, crude rates per 1,000 live births, and ranks are shown. Source: https://wonder.cdc.gov/controller/saved/D76/D313F351

© 2023 Flaxman S et al. *JAMA Network Open.*

eTable 2C<br>Ages: 0-364 days			
**10 Leading Causes of Death (Infants)**	**Deaths**	**Crude Rate (per 1000 live births)**	**Rank**
#Congenital malformations, deformations and chromosomal abnormalities (Q00-Q99)	4,301	1.1	1
#Disorders related to short gestation and low birth weight, not elsewhere classified (P07)	3,445	0.9	2
#Accidents (unintentional injuries) (V01-X59)	1,266	0.3	3
#Sudden infant death syndrome (R95)	1,248	0.3	4
#Newborn affected by maternal complications of pregnancy (P01)	1,245	0.3	5
#Newborn affected by complications of placenta, cord and membranes (P02)	742	0.2	6
#Bacterial sepsis of newborn (P36)	603	0.2	7
#Respiratory distress of newborn (P22)	424	0.1	8
#Diseases of the circulatory system (I00-I99)	406	0.1	9
#Necrotizing enterocolitis of newborn (P77)	354	0.1	10

Supplementary Table 2(c). Leading causes of death from the rankable causes on the NCHS 130 Selected Causes of Infant Death List, for infants 0-364 days old in 2019 in the US ranked. There were 153 Covid-19 deaths (July 1, 2021-August 31, 2022) for this age group. Deaths, crude rates per 1,000 live births, and ranks are shown. Source: https://wonder.cdc.gov/controller/saved/D76/D313F352

© 2023 Flaxman S et al. *JAMA Network Open.*
