# Supplementary material for: Assessment of COVID-19 as the Underlying Cause of Death Among Children and Young People Aged 0 to 19 Years in the US
Source: JAMA Netw Open. 2023 Jan 30;6(1):e2253590. doi: 10.1001/jamanetworkopen.2022.53590 (PMC9887489; doi:10.1001/jamanetworkopen.2022.53590)
Supplement: Supplement 2. — Data Sharing Statement [file jamanetwopen-e2253590-s002.pdf]

Data Sharing Statement

Flaxman. Assessment of COVID-19 as the Underlying Cause of Death Among Children and Young People Aged 0 to 19 Years in the US. *JAMA Netw Open*. Published January 30, 2023. doi:10.1001/jamanetworkopen.2022.53590

Data
Data available: Yes
Data types: Deidentified participant data, Data dictionary
How to access data: https://github.com/MLGlobalHealth/covid19pediatric
When available: With publication

Supporting Documents
Document types: Statistical/analytic code
How to access documents: https://github.com/MLGlobalHealth/covid19pediatric
When available: With publication

Additional Information
Who can access the data: Publicly available
Types of analyses: Analysis must follow CDC WONDER rules: https://wonder.cdc.gov/mcd-icd10-provisional.html
Mechanisms of data availability: Without investigator support
Any additional restrictions: none
